# Quantitative analysis of tobacco blending proportions based on hyperspectral imaging and data fusion

**DOI:** 10.3389/fpls.2025.1736546

**Published:** 2026-01-15

**Authors:** Yifan Jiang, Qinlin Xiao, Xudong Huang, Ruifang Gu, Jing Wen, Xixiang Zhang, Yang Liu, Li Li, Xiaojing Chen, Juan Yang, Yong He

**Affiliations:** 1 College of Biosystems Engineering and Food Science, Zhejiang University, Hangzhou, China; 2 Technology Center, China Tobacco Sichuan Industrial Co., Ltd., Chengdu, China; 3 School of Opto-Electronic Engineering, Changchun University of Science and Technology, Changchun, China; 4 College of Electrical and Electronic Engineering, Wenzhou University, Wenzhou, China

**Keywords:** blending proportions, hyperspectral imaging, multispectral fusion, quantitative analysis, tobacco

## Abstract

The rapid and accurate detection of tobacco blending proportions is essential for quality control in the tobacco industry. This study proposes a method for the quantitative analysis of tobacco components based on multispectral fusion, integrating visible-near-infrared (Vis-NIR) and near-infrared (NIR) spectral data. The method employs the minimum covariance determinant (MCD) for anomaly detection and constructs a quantitative model using partial least squares regression (PLSR). The experimental data comprise two matrices of dimensions 400 × 90 and 220 × 90, each containing 90 samples. Experimental results demonstrate that multispectral fusion significantly improves the model’s quantitative analysis performance compared to using a single spectrum. The adopted preprocessing strategy effectively reduces noise interference and enhances feature extraction capability. When predicting tobacco silk content, the fused spectral model achieved the highest prediction accuracy with R^2^ of 0.8873. The innovation of this study lies in the proposed multispectral data optimization fusion and preprocessing strategy, which facilitates rapid detection of tobacco constituents and offers an optimal and efficient method. This approach provides a reliable technical solution and advances spectral detection technology in the tobacco and related industries.

## 1 Introduction

As a globally significant cash crop, tobacco plays a pivotal role in agricultural economies, industrial manufacturing, and international commerce. Southwest China has established itself as the nation’s premier production base for premium flue-cured tobacco, due to its exceptional climatic conditions and ideal summer growing environment (Yao et al., 2024). Within this context, tobacco blend ratio testing represents a fundamental quality control measure for maintaining consistent sensory characteristics in cigarette products (Wu et al., 2022). Different tobacco varieties, growing regions, and quality grades demonstrate marked variations in aromatic profile, flavor intensity, and smoking characteristics (Niu et al., 2022). Critically, slight deviations in blending proportions can result in noticeable batch-to-batch inconsistencies - manifested as diminished fragrance, undesirable aftertastes, or increased irritation - all of which directly compromise the consumer experience. Maintaining precise blend ratios is essential for preserving the distinctive sensory signature of each cigarette, ensuring every unit faithfully reproduces the brand’s intended flavor architecture. Beyond quality assurance, optimized blending protocols enable efficient raw material utilization by preventing both the overuse of premium leaves and excessive incorporation of lower-grade tobaccos, while simultaneously guaranteeing compliance with both manufacturing specifications and regulatory standards. Through rigorous monitoring and control of blending uniformity, manufacturers can deliver product consistency at scale, meeting consumer expectations for sensory performance while strengthening brand equity in an increasingly competitive marketplace.

At present, the conventional methodologies employed in the tobacco industry for the determination of components primarily encompass chemical analysis by chromatographic techniques and solvent extraction, as well as physical detection by microscopic observation (Chen et al., 2021; Losso et al., 2022; Li et al., 2024a). While these approaches are indeed capable of determining critical components such as nicotine, sugar, and tar in tobacco, their limitations are equally pronounced. Chemical analysis typically necessitates complex pretreatment processes, which are both time-consuming and destructive (Losso et al., 2022). Physical methods, on the other hand, are susceptible to subjective interpretation and lack the requisite precision. Moreover, these methods struggle to support real-time monitoring, trace components, or complex mixtures of limited resolution. Additionally, the equipment costs and maintenance requirements are high, impeding production efficiency and the necessity for precise control. Therefore, hyperspectral imaging technology and similar emerging technology fields are rapidly becoming the focus of industrial research.

Hyperspectral imaging technology (HSI) demonstrates unique advantages in the field of quality inspection (Özdoğan et al., 2021; Ahmed et al., 2024; Medina–García et al., 2025). It combines spectral analysis with image recognition to simultaneously capture spectral and spatial distribution data of the target object, thereby achieving a “spectral-image integration” inspection effect (Zhao et al., 2023). Additionally, its ultra-high spectral resolution and rich spatial information enable it to more accurately identify and locate different components within a sample. In terms of detection performance, HSI offers fast, non-destructive, high-sensitivity detection with real-time monitoring capabilities. These characteristics make it highly valuable in applications such as agricultural product quality grading (Gao and Xie, 2024) and impurity identification (Jia et al., 2024; Nargesi et al., 2024; Shiny et al., 2024; Fei et al., 2025). Additionally, with the development of machine learning algorithms, the processing efficiency and analysis accuracy of hyperspectral data have been significantly improved, providing new technical pathways for quality detection in complex scenarios. Currently, this technology has been successfully applied in multiple fields such as food component visualization analysis (Shi et al., 2024; Seong et al., 2025) and drug uniformity assessment (Belay et al., 2021; Rocha de Oliveira and de Juan, 2021), with its detection effectiveness widely validated. Tian et al. evaluated the effect of drying processes on the anthocyanin and moisture content of purple sweet potatoes, using HSI to estimate and generate a visual map of the distribution patterns in processed purple sweet potatoes (Tian et al., 2021). Xie et al. developed a method using 750 nm/900 nm ratio imaging combined with machine vision to accurately quantify spice uniformity in cooked meat products (Xie et al., 2022a).

However, the adoption of spectral technologies in tobacco remains at a developing stage. As summarized in **Table 1**, current research indicates that NIR spectroscopy is the most widely used technique, offering reliable quantitative prediction of key constituents such as nicotine, sugars, and moisture in tobacco. Non-imaging spectroscopic methods dominate present applications, while hyperspectral imaging is still relatively underexplored in this specific domain, despite its potential for spatially resolved chemical mapping. Existing technologies continue to encounter certain limitations. Conventional spectroscopy captures limited molecular information per data point, insufficient for complex component characterization. This limitation renders it challenging to provide a comprehensive characterization of complex components. The utilization of single spectral data invariably results in suboptimal model generalization capability, thereby compromising the precision of concurrent detection of multiple indicators (Liang et al., 2023). Concurrently, within the manufacturing facility, factors such as tobacco humidity, granularity variations, equipment vibration, and fluctuations in environmental temperature and humidity levels contribute to the introduction of spectral noise, thereby diminishing the signal-to-noise ratio (Tiplady et al., 2019). The existing algorithms have been demonstrated to lack sufficient robustness in the presence of strong noise backgrounds, resulting in substantial fluctuations in detection outcomes. The impact of data processing, spectral processing denoising, baseline correction, and other steps on model performance is significant. The selection of existing methods is largely dependent on the experience of experts and lacks adaptive optimization strategies, which can lead to overfitting or information loss (Shi et al., 2023).

**Table 1:** Summary of prior studies on spectral analysis for tobacco.

Spectral type	Imaging	Main detection index	Methodology	Research progress	References
NIR	No	Stem ratio within tobacco blend	Through the preprocessing of the spectrum and the partial least squares method	Achieved accurate stem ratio prediction (error <3.88%) and enabled uniformity assessment for single cigarettes	(Wu et al., 2022)
NIR	No	71 chemical components in tobacco, including routine chemicals, polyphenolic compounds, and organic acids.	A Just-In-Time Learning-integrated Partial Least Squares (JIT-PLS) modeling strategy was employed on a large spectral database to dynamically select optimal local calibration subsets for each prediction sample.	The JIT-PLS models significantly outperformed traditional PLS, substantially improving prediction accuracy (R² up to 0.996) for 67 major components across tobacco leaf and cigarette samples	(Liang et al., 2022)
NIR	No	Cigarette brand identification for authenticity verification.	Near-infrared spectroscopy coupled with optimized machine learning, featuring spectral preprocessing (e.g., Savitzky–Golay 1st derivative) and variable selection (UVE) to refine input data, followed by classification models including SVM, MLP, and CNN.	Achieved perfect authentication (100% accuracy) on test sets with optimized models	(Cao et al., 2025)
NIR and MIRS	No	Total nicotine, total sugar, reducing sugar, and total nitrogen content in tobacco.	Near-infrared and mid-infrared spectral data were fused using two techniques (variable-level fusion and data-level fusion) combined with multiple wavelength selection algorithms (SPA, CARS, iMWPLS, etc.) to build Partial Least Squares Regression models.	The optimized fusion models significantly improved prediction accuracy for all four components, with RMSEP notably reduced	(Wang et al., 2024)
NIR	No	Ten sensory attributes of flue-cured tobacco for quality grading.	Near-infrared spectroscopy combined with a novel Polarization-Standard Filtering (PSF) preprocessing operator, followed by Support Vector Machine (SVM) modeling and sliding-window occlusion for spectral interpretation.	PSF preprocessing significantly improved model discriminability, achieving 99.7% accuracy for 7-level quality grading and high accuracies (up to 98.3%) for multiple sensory attributes, enabling objective and nondestructive tobacco grading.	(Ye et al., 2025)
Temporal Hyperspectral Imaging	Yes	Early detection of mold development in tobacco leaves	Dynamic spectral features were extracted from time-series hyperspectral images, and a Conditional Wasserstein GAN with Gradient Penalty (CWGAN-GP) was employed to address class imbalance for model training.	The method effectively identified early spectral changes associated with mold, providing a viable approach for early warning and real-time monitoring of tobacco leaf mold.	(Li et al., 2025)
NIR Hyperspectral Imaging	Yes	Discrimination of four cut tobacco components (lamina cut tobacco, stem cut tobacco, reconstituted tobacco cut tobacco, expanded cut tobacco)	Spectral preprocessing: second-order derivative combined with smoothing filter; Feature wavelength selection: second-order derivative and Successive Projections Algorithm (SPA); Discrimination modeling: Support Vector Machine (SVM) and K-Nearest Neighbors (KNN).	The SVM model based on characteristic wavelengths achieved a discrimination accuracy of 86.97% for sample components. This demonstrated the feasibility of applying hyperspectral imaging technology to the discrimination of cut tobacco components in formulations.	(Mei et al., 2021)

To address the aforementioned challenges, this study proposes a hyperspectral imaging-based framework for quantitative analysis of blended tobacco composition. The framework is centered around two core scientific problems: improving the accuracy of simultaneous multi-component analysis and enhancing anti-interference capability in complex industrial environments. Its key innovations include a feature-level fusion strategy for complementary visible-near infrared (Vis-NIR) and near infrared (NIR) spectra data and an anti-interference modeling method that integrates the minimum covariance determinant (MCD) anomaly detection algorithm with partial least squares regression (PLSR). Experimental results demonstrate that the proposed spectral fusion modeling approach improves the prediction accuracy of tobacco silk components by 14.83% compared to single-spectral methods, achieving an R^2^ value of 0.8873. This method not only significantly enhances data processing efficiency and reproducibility but also provides a new perspective for intelligent quality detection in the tobacco industry, thereby supporting its transition toward digitalization and automation.

## 2 Materials and methods

### 2.1 Experimental materials

**Figure 1:**
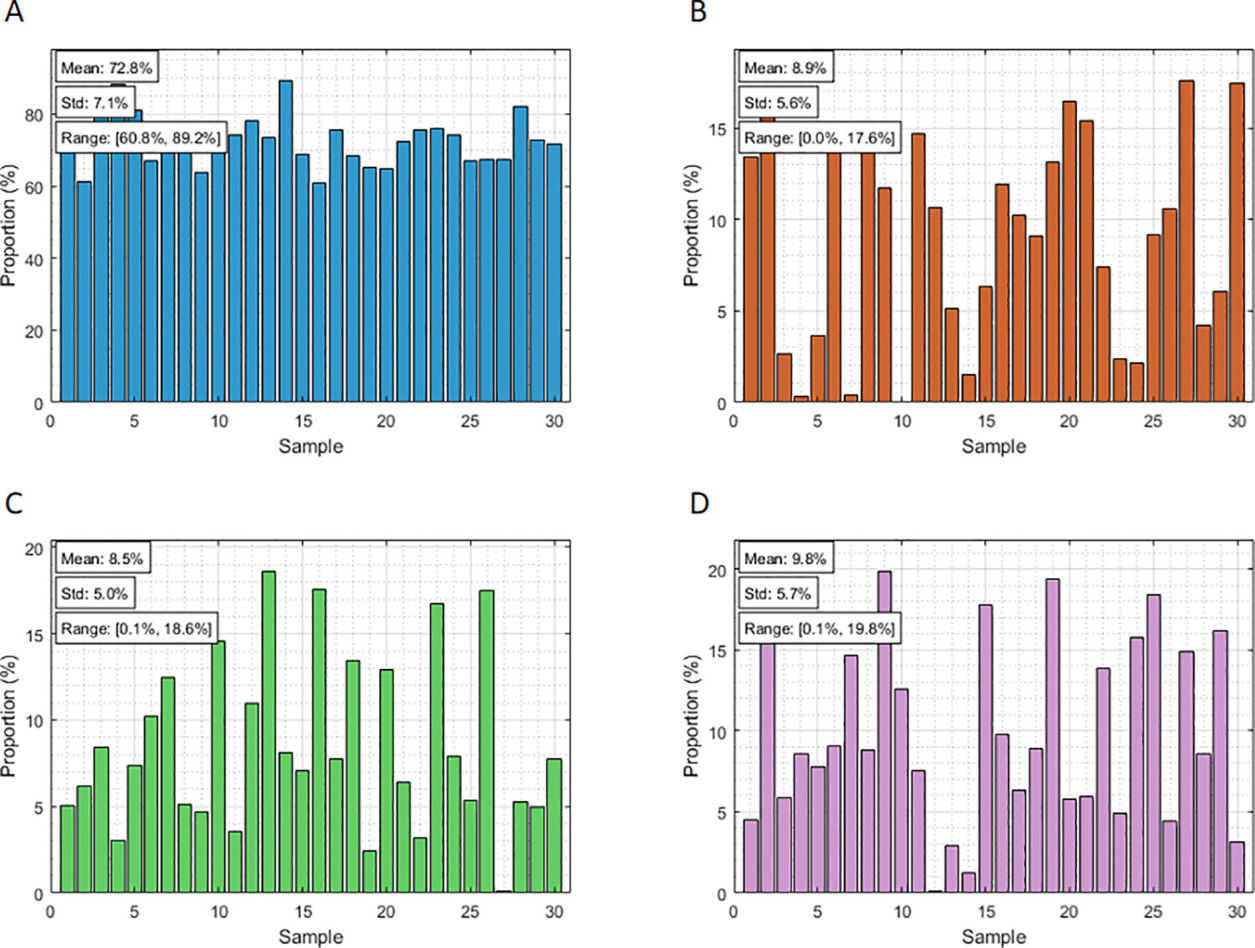
Distribution of four tobacco components: **(A)** tobacco silk proportion distribution; **(B)** cut stem proportion distribution; **(C)** fermented cut stem proportion distribution; **(D)** expanded tobacco silk. In all subplots, the x-axis corresponds to sample ID (1–30), and the y-axis represents the proportion of each component within the corresponding sample (%).

The experimental samples utilized in this study were provided by China Tobacco Sichuan Industrial Co., Ltd., and consisted of four tobacco components with divergent physicochemical properties, including (1) tobacco silk (60-100%); (2) cut stem (0-20%); (3) fermented cut stem (0-20%); and (4) expanded tobacco silk (0-20%). These components were obtained directly from the production line, with their respective moisture contents measured as follows: approximately 13.3% for tobacco silk, 12.5% for cut stem, 12.5% for fermented cut stem, and in the range of 12.0–12.6% for expanded tobacco silk. A total of 30 distinct blended tobacco samples were meticulously prepared, each with a standard mass of 20 g. To ensure diversity and representativeness, the specific blending proportions were determined using a constrained random number generation algorithm. The algorithm was designed to independently generate the proportion of each component within its respective allowable range while constraining the sum of all components to exactly 100%. This approach ensured uniform coverage across the defined proportion space and prevented over-representation of extreme or central proportion combinations. The resulting distribution of the four components is illustrated in **Figure 1**. During sample preparation, each component was meticulously weighed using a precision electronic balance.

The blended tobacco was accurately weighed and poured into a box, shaken, thoroughly mixed, and then evenly laid on black A4 cardboard for hyperspectral scanning. To ensure consistency, each sample was carefully spread to form a thin layer uniform thickness without exposing the substrate. To minimize the effects of mixing inhomogeneity and spatial distribution variations, each proportion was subjected to triple validation: after each scanning cycle, the sample was collected, remixed, and re-spread uniformly before the next scan. This process was repeated three times per blending ratio, resulting in a total of 90 samples for subsequent processing and analysis.

### 2.2 Data acquisition

**Figure 2:**
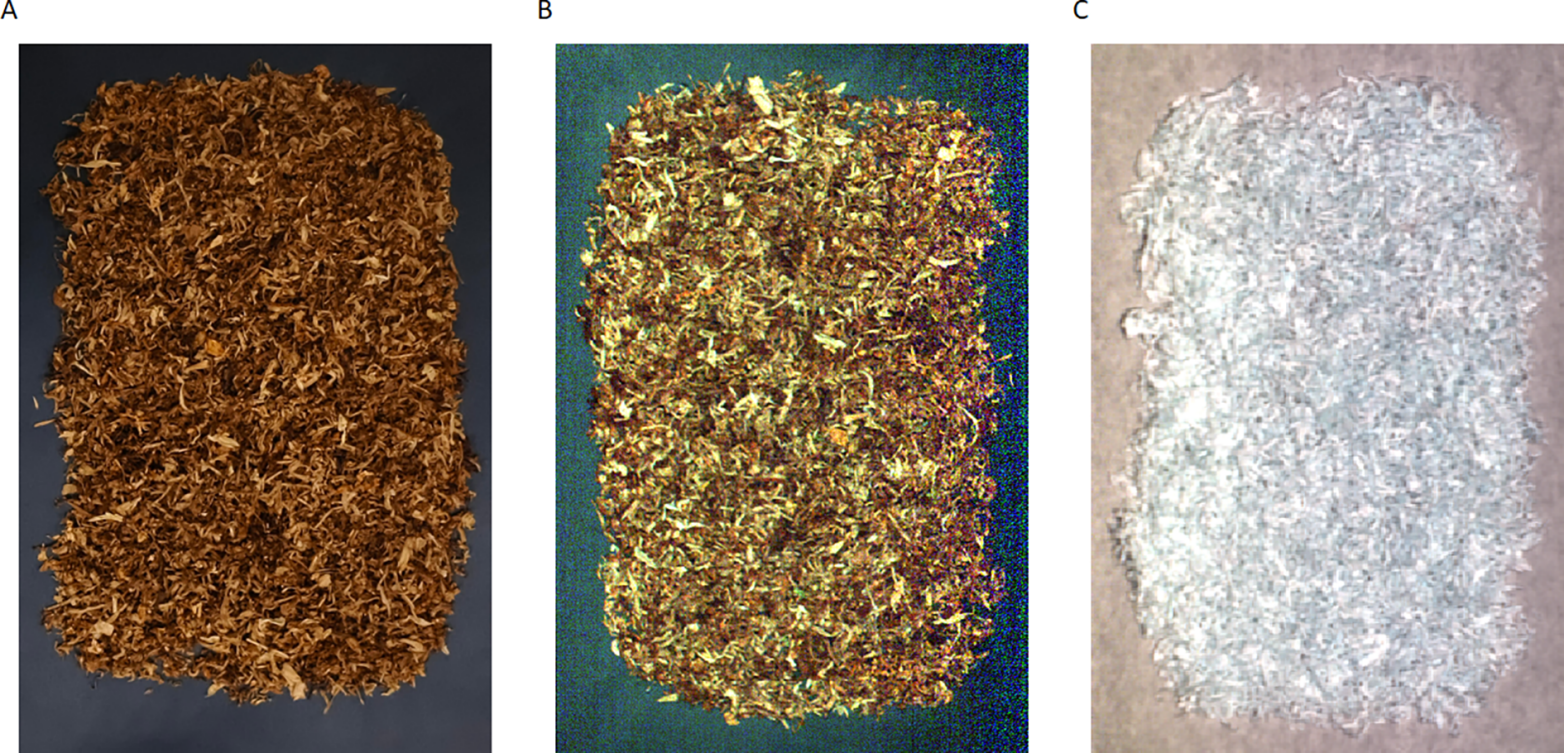
Representative tobacco images under different imaging modalities: **(A)** RGB image; **(B)** Vis-NIR image; **(C)** NIRimage.

A hyperspectral imaging system was utilized in the study to collect spectral data of blended tobacco with varying blending ratios. The system comprises a visible near-infrared hyperspectral imager, a near-infrared hyperspectral imager, a CCD camera, optical lenses, two 150W tungsten-halogen line light sources, an electronically controlled mobile platform, and a computerized control terminal. The visible near-infrared imaging spectrometer is the ImSpectorV10E model (Specim, Spectral Imaging Ltd., Oulu, Finland), which is capable of acquiring hyperspectral images within the 400–1000 nm band spectrum, with a spectral resolution of 2.8 nm. For Vis-NIR acquisition, the translation stage speed is set to 7.5 mm/s, the camera exposure time is 21.231 ms, and the working distance between the sample and lens is 37.1 mm. The near-infrared imaging spectrometer is the ImSpector N17E model (Specim, Spectral Imaging Ltd., Oulu, Finland), operating in the range of 900–1700 nm band with a spectral resolution of 5 nm. For NIR acquisition, the translation stage speed is 9.84 mm/s, the exposure time is 30 ms, and the working distance is 32.7 mm. In order to mitigate the impact of external environmental light sources on the spectral acquisition process, a black box is positioned externally within the spectral imaging acquisition instrument and the supporting equipment. When the system works, the computer control terminal initiates the startup of each system component. The motor drives the samples along the conveyor belt, the imaging spectrometer with the camera and lens acquires hyperspectral images of the samples, and the acquired image data is transmitted to the computer control terminal, thereby completing the data acquisition process. The captured image is shown in **Figure 2**.

Significant noise is introduced due to light intensity fluctuation and sensor dark current during the acquisition process, which leads to degradation of hyperspectral image quality and directly affects data accuracy. To address this issue, this study used a black-and-white correction algorithm to process the raw data. The calibration was systematically performed before each measurement session using a high-reflectivity white reference board and a dark background image captured with the lens covered. This procedure was repeated every 30 minutes throughout the acquisition process to maintain calibration stability. The equation is shown in Equation 1:

(1) $$R=(I_{raw}-I_{dark})/(I_{ref}-I_{dark})$$

where R means the corrected sample image; $I_{raw}$ stands for the original image of the sample; $I_{dark}$ denotes the dark background correction image; and $I_{ref}$ represents the whiteboard correction image.

### 2.3 Methods of analysis

#### 2.3.1 PLSR regression modeling process

PLSR represents a robust multivariate analysis method particularly suited for high-dimensional spectral data analysis. Its demonstrated efficacy in small-sample, high-dimensional scenarios has led to widespread adoption in agricultural and food science applications of near-infrared spectroscopy (Arnese-Feffin et al., 2024; Li et al., 2024b; Zheng et al., 2024). The method simultaneously decomposes the predictor variable matrix X and the response variable matrix Y into a series of orthogonal latent variables (LVs), which are linear combinations of the original variables. The extraction process is based on the criterion of maximizing the covariance between X and Y (Fernández-Habas et al., 2022; Li et al., 2024c). In order to achieve the objective of specific modeling, the raw spectral data must first undergo a series of processing steps. The first step is to preprocess the spectra to eliminate background noise and baseline drift. Subsequently, the optimal number of LVs was subsequently determined using Leave-One-Out Cross-Validation (LOOCV), with the first local minimum of the Root Mean Square Error of Cross-Validation (RMSECV) as the selection criterion (Deng et al., 2024).

In order to enhance the model’s performance, Spectral preprocessing methods, including Savitzky-Golay smoothing (SG), derivative, standard normal variate (SNV), and multiplicative scatter correction (MSC), were employed to eliminate scattering effects, noise interference, and baseline drift. This process could enhance the prediction accuracy and model robustness of the regression task (Zhu et al., 2025). For different spectral data and target variables, multiple preprocessing methods were compared to determine the optimal approach for each case. In order to enhance the prediction accuracy, robustness, and interpretability of the PLSR model, a variety of wavelength selection methods were employed after spectral preprocessing. These include Variable Importance in Projection (VIP), Successive Projections Algorithm (SPA), and Competitive Adaptive Reweighted Sampling (CARS), which are utilized to identify a limited number of key wavelengths that exhibit a strong correlation with the target variables (Xie et al., 2022b). The implementation of these methods ensures the elimination of wavelengths with a low signal-to-noise ratio or those that are irrelevant to the target variables. This process also serves to mitigate the impact of redundant wavelengths, thereby enhancing the overall performance of the model. This step offers a dual benefit of enhancing prediction accuracy and facilitating physical interpretability for quantitative spectral analysis.

To rigorously evaluate model performance and generalizability within the available dataset, a full cross-validation procedure was adopted for all PLSR models, thereby forgoing the use of a separate hold-out test set. This approach is standard in spectroscopic calibration when sample availability is constrained, as it allows for a robust estimation of predictive accuracy by iteratively using the entire dataset for both training and validation.

#### 2.3.2 Outlier detection

The presence of outliers can have multiple negative impacts on statistical analysis and regression modeling, including but not limited to: undermining the assumption of sample normality, increasing the variability of results and estimation errors, reducing the efficacy of hypothesis testing, introducing systematic bias, and weakening the predictive accuracy of the model (Zheng et al., 2024). Therefore, when constructing multiple regression models, the identification and treatment of outliers is a critical aspect of ensuring reliable data.

In the context of PLSR modeling, the identification of outliers constitutes a pivotal step in ensuring the robustness of the model. In this study, MCD algorithm was employed for outlier detection. As a robust statistical method, MCD operates by identifying the optimal subset of h samples (where h = 0.75n from total n samples) that minimizes the determinant of their covariance matrix (Huang et al., 2024). This approach effectively captures the data’s core distribution while providing robust estimates of both location parameters and scatter matrix, making it particularly suitable for detecting anomalies in complex spectral datasets. The selected subset represents the most tightly clustered portion of the data, with observations deviating from this core group being reliably identified as outliers (Hussain et al., 2024).

In addition, since the MCD algorithm is most effective for low-dimensional data, we first applied PCA to reduce the dimensionality of the spectral data (Alrawashdeh et al., 2024). This step ensures that the number of samples in the input matrix exceeds the number of variables, making MCD-based outlier detection feasible. The variance explained ratio in PCA is a pivotal indicator of the proportion of original data retained by each principal component. The sum of the variance explained ratios of the first m principal components is denoted as the cumulative variance explained ratio, which reflects the aggregate proportion of original data retained.

#### 2.3.3 Evaluation indicators

In order to assess the predictive performance of the established model, the following two key indicators were used for quantitative analysis. Coefficient of Determination (R^2^) was used to measure the model’s ability to explain the variation in the data, with a value ranging from 0 to 1. The closer R^2^ is to 1, the better the model is fitted. Root Mean Square Error (RMSE) was used to assess the prediction accuracy of the model, calculating the deviation between the predicted value and the measured value, the smaller the RMSE is, the better the prediction ability of the model is. R² is given by Equation 2, and RMSE is defined by Equation 3:

(2) $$R^{2}=1-\frac{\sum_{i=1}^{n}{\left(y_{i}-{\hat{y}}_{i}\right)}^{2}}{\sum_{i=1}^{n}{\left(y_{i}-\bar{y}\right)}^{2}}$$

(3) $$RMSE=\sqrt{\frac{1}{n}\sum_{i=1}^{n}{\left(y_{i}-{\hat{y}}_{i}\right)}^{2}}$$

where y is the concentration value attribute of the sample, $\hat{y}$ is the concentration value attribute predicted by the model, and $\bar{y}$ is the mean value of the concentration value attribute of the sample.

## 3 Results and discussion

### 3.1 Raw spectral modeling results

#### 3.1.1 Raw spectra of Vis-NIR and NIR

**Figure 3:**
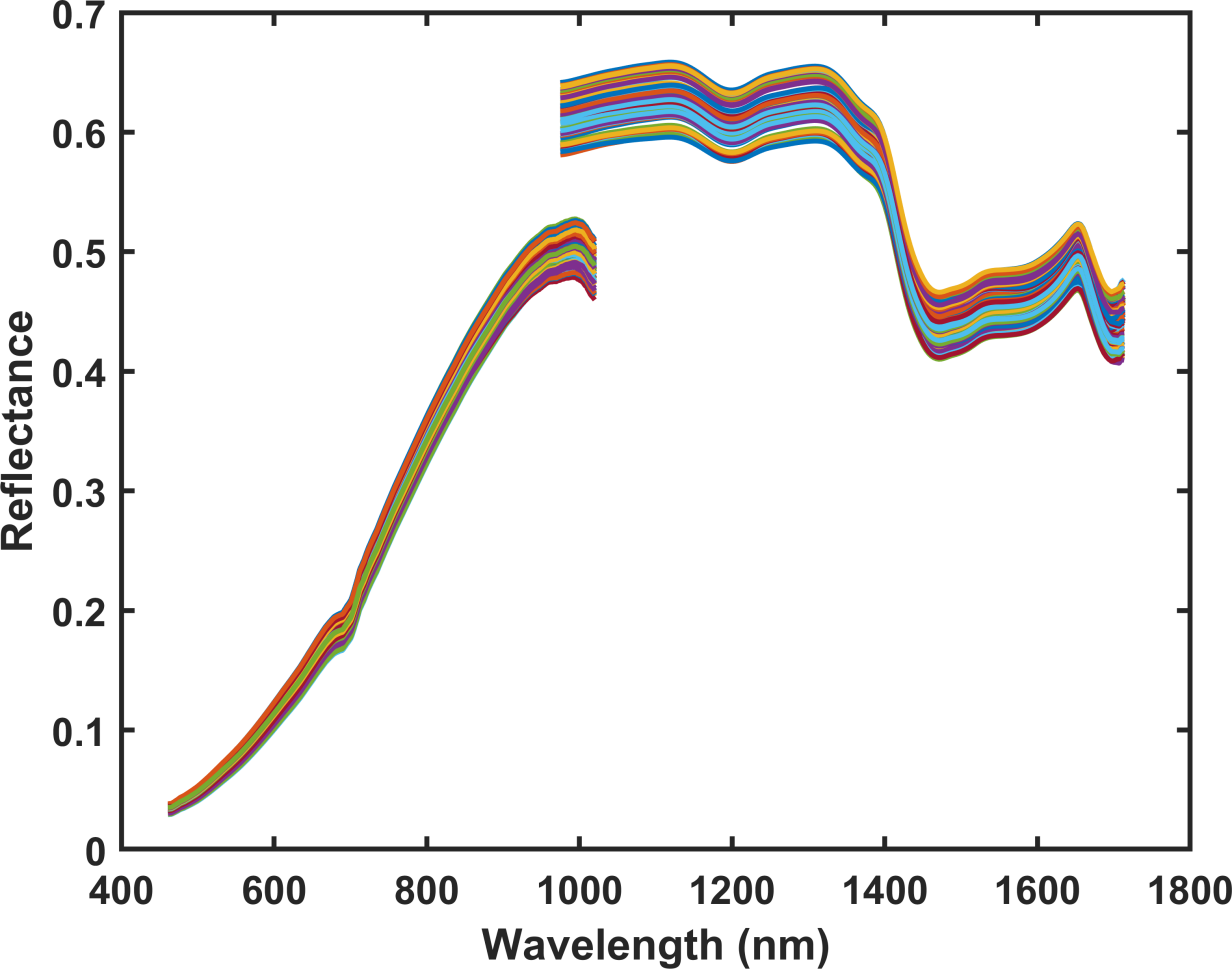
The original Vis-NIR spectra (460–1020 nm) and NIR spectra (975–1713 nm).

To mitigate instrument noise, optimal spectral intervals were selected from the independently acquired datasets: the Vis-NIR spectra (460–1020 nm, 400 wavelengths) and the NIR spectra (975–1713 nm, 220 wavelengths). The final experimental data comprise two matrices of dimensions 400 × 90 and 220 × 90, each containing 90 samples. The reflectance spectra of tobacco samples, acquired from Vis-NIR and NIR ranges, are presented in **Figure 3**. In addition, although the overlapping region (975–1020 nm) shows noticeable reflectance differences between the Vis–NIR and NIR instruments due to their distinct detector responses, this region was retained in the subsequent wavelength-selection stage so that the AutoPF framework (Chen et al., 2025) and its data-driven algorithms could objectively determine whether these wavelengths contain useful information.

The spectral curve of tobacco exhibits characteristic changes. The Vis-NIR spectrum of tobacco shows an overall increase in reflectance. A key absorption valley at 650–700 nm is due to chlorophyll absorbing red light. In the NIR region, the spectrum fluctuates downward. The distinct absorption trough at 1450–1500 nm corresponds to water and cellulose (O-H and C-H vibrations). The characteristic peak at 1650–1700 nm mainly reflects the presence of nicotine (N-H vibration) and polyphenolic compounds (Workman and Weyer, 2007). These specific wavelength bands effectively map onto the chemical fingerprints of tobacco’s major components—chlorophyll, water, cellulose, nicotine, and polyphenols. Therefore, they serve as important indicators for analyzing tobacco composition and differentiating blending materials.

#### 3.1.2 Results of quantitative analysis of tobacco blending components

Spectral fusion of Vis-NIR and NIR can provide more comprehensive and complementary information. Vis-NIR mainly contains color information and other features related to electron jumps, while NIR is more related to the octave and merge frequencies of molecular vibrations. Fusing the two can provide more comprehensive information about the substance, especially when the substance has both color changes and chemical composition changes. In quantitative analysis, the use of spectral fusion strategy can enhance the model robustness. A single spectrum may be more affected by noise or interference in some cases, while the fused data can reduce this effect by the information of multiple ranges, and improve the stability and prediction ability of the model.

In this study, Vis-NIR and NIR and their fusion spectra were used to quantitatively analyze the four adulterant components in tobacco samples. The regression results of the PLSR model are shown in **Table 2**. For the quantitative analysis of adulterants in tobacco samples, namely tobacco silk, fermented cut stem, and expanded tobacco silk, the modeling accuracy of Vis-NIR and NIR fusion spectroscopy is higher than that of Vis-NIR or NIR alone, with R^2^ values of 0.7727, 0.7046, and 0.6676, respectively. This suggests that the prediction accuracies of quantitative analyses can be enhanced by fusing Vis-NIR and NIR through the multidimensional complementary information and the synergistic cross-range features. In the context of quantitative analysis of cut stem, a notable phenomenon emerges: Vis-NIR accuracy exhibits a marginal superiority over fused spectra. This observation can be attributed to the diminished correlation between the target variable, cut stem, and NIR information. Consequently, extraneous noise variables are introduced into the fused spectra, leading to a reduction in prediction accuracy when compared to the accuracy of single Vis-NIR.

**Table 2:** Results of PLSR models for the original spectra.

Doping component	Spectral data	LVs	R^2^	RMSECV	RPD
Tobacco silk	Vis-NIR	11	0.7218	0.0370	1.9066
	NIR	14	0.7717	0.0335	2.1048
	Vis-NIR+NIR	**18**	**0.7727**	**0.0334**	**2.1093**
Cut stem	Vis-NIR	**10**	**0.5786**	**0.0360**	**1.5492**
	NIR	9	0.5267	0.0382	1.4617
	Vis-NIR+NIR	10	0.5774	0.0361	1.5469
Fermented cut stem	Vis-NIR	13	0.6707	0.0280	1.7523
	NIR	17	0.6567	0.0286	1.7162
	Vis-NIR+NIR	**14**	**0.7046**	**0.0265**	**1.8503**
Expanded tobacco silk	Vis-NIR	11	0.4644	0.0410	1.3741
	NIR	10	0.6120	0.0349	1.6163
	Vis-NIR+NIR	**19**	**0.6676**	**0.0323**	**1.7442**

Bold values indicate the optimal PLSR model within each component.

### 3.2 Rejects anomalous samples by MCD

#### 3.2.1 Characteristics of the distribution of anomalous samples

In this study, PCA was applied to reduce the dimensionality of the spectral data before performing outlier detection using the MCD algorithm. The selection of principal components was based on cumulative variance contribution. The first two principal components (PC1-PC2) account for more than 97% of the total variance for all three spectral datasets (99.67% for Vis-NIR, 99.10% for NIR, and 97.68% for the fusion spectra of Vis-NIR and NIR). The variance contributions of PC3-PC5 are all below 2%, indicating that these components mainly reflect noise or minor fluctuations. Therefore, retaining only the first two principal components ensures that almost all meaningful spectral information is preserved while avoiding noise-dominated redundant components that may reduce the robustness of MCD. Moreover, MCD achieves more stable covariance estimation in a lower-dimensional space, and the rapid decline in variance contribution beyond PC2 provides a consistent criterion for the three spectral datasets.

**Figure 4:**
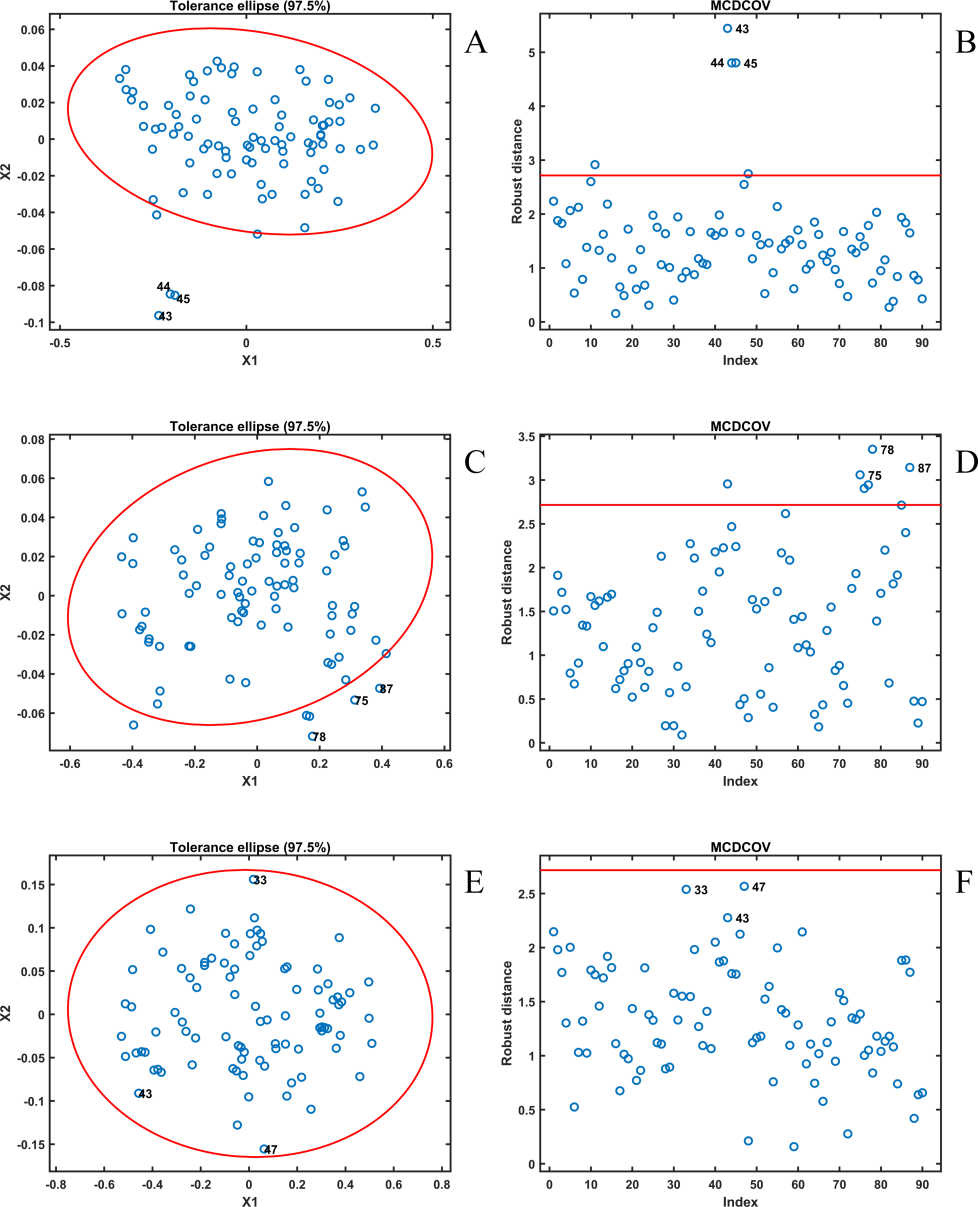
Results in MCD: **(A)** Data distribution of Vis-NIR dataset; **(B)** Robust distance of Vis-NIR dataset; **(C)** Data distribution of NIR dataset; **(D)** Robust distance of NIR dataset; **(E)** Data distribution of fusion spectra dataset; **(F)** Robust distance of fusion spectra dataset. The red ellipse in **(A, C, E)** represents the 97.5% tolerance ellipse, and the red horizontal line in **(B, D, F)** is the threshold for determining outliers. Points that fall outside the ellipse or above the red line will be marked as outliers.

For the Vis–NIR dataset, the cumulative variance explained by PC1-PC2 reached 99.67%, confirming that these components retain nearly all of the original spectral information. The results of Vis-NIR using MCD to reject abnormal samples are shown in **Figure 4**, where the red ellipse in **Figure 4A** represents the 97.5% tolerance ellipse. From **Figure 4A**, it can be seen that five observations were excluded from the tolerance ellipse, with samples 43, 44, and 45 having data that are clearly different from the distribution of the overall sample, and these samples can affect the precision of subsequent quantitative analyses. Similarly, it can be seen in **Figure 4B** that the distance of these five observations to the robust subset in the MCD was greater than the cutoff value, and thus they were judged to be anomalous samples to be rejected and not involved in the subsequent quantitative analysis.

For the NIR spectra dataset, after PCA applied, the cumulative variance explained by the first two principal components reaches 99.10%, so the same first two principal components of the NIR spectra can be used for MCD anomaly sample rejection. In **Figure 4C**, there are 6 observations that fall outside the tolerance ellipse and are considered to be inconsistent with the distribution of the overall sample. And similarly, these 6 observations were considered to be anomalous samples in **Figure 4D** as their robust distances were larger than the cutoff value. These anomalous samples were removed to improve the accuracy of the subsequent quantitative analysis.

The fusion spectra dataset of Vis-NIR and NIR were subjected to PCA downscaling, and the cumulative variance explained by the first two principal components amounted to 97.68%. And similarly, these two principal components were used for MCD to reject the anomalous samples. As shown in **Figure 4E**, all observations fall within the tolerance ellipse, which indicates that for the fusion spectra of Vis-NIR and NIR, all samples come from the same distribution and there are no anomalous samples. It can also be seen from **Figure 4F** that the distances of all samples to the robust subset in the MCD are less than the cutoff value. Thus all 90 samples were used for subsequent quantitative analysis.

#### 3.2.2 Quantitative analysis results after removal of outliers

The MCD removal of anomalous samples was performed on Vis-NIR dataset and NIR dataset, and five and six spectral data were removed, leaving the number of samples at 85 and 84, respectively. The fusion spectra of Vis-NIR and NIR did not have any obvious anomalous samples, so the number of samples remained at 90. The modeling results after using MCD for the raw spectra to remove outliers are shown in **Table 3**.

**Table 3:** Results of PLSR models after MCD eliminates abnormal samples.

Doping component	Spectral data	Sample size	LVs	R^2^	RMSECV	RPD
Tobacco silk	Vis-NIR	85	11	0.7613	0.0335	2.0587
	NIR	**84**	**9**	**0.8143**	**0.0308**	**2.3343**
	Vis-NIR+NIR	90	18	0.7727	0.0334	2.1093
Cut stem	Vis-NIR	85	10	0.5516	0.0375	1.5022
	NIR	84	7	0.5690	0.0375	1.5324
	Vis-NIR+NIR	**90**	**10**	**0.5774**	**0.0361**	**1.5469**
Fermented cut stem	Vis-NIR	85	13	0.6670	0.0281	1.7432
	NIR	84	16	0.6631	0.0274	1.7332
	Vis-NIR+NIR	**90**	**14**	**0.7046**	**0.0265**	**1.8503**
Expanded tobacco silk	Vis-NIR	85	7	0.5574	0.0370	1.5120
	NIR	84	10	0.6408	0.0331	1.6785
	Vis-NIR+NIR	**90**	**19**	**0.6676**	**0.0323**	**1.7442**

Bold values indicate the optimal PLSR model within each component.

The regression results of the PLSR model were improved after the five anomalous samples in Vis-NIR dataset were excluded, and when the target variable was expanded tobacco silk, the R^2^ was improved from 0.4644 to 0.5574, and the regression accuracy was improved by 20.03%, and the prediction results of the PLSR model did not change much when the target variables were tobacco silk, cut stem, and fermented cut stem. This may be due to the presence of anomalous samples in spectral bands that are not related to these three target variables, which depend on other stable bands, resulting in little change in the accuracy of the PLSR model after the removal of the anomalous samples. The six anomalous samples in the NIR were removed from the MCD and modeled using the remaining spectral data, and for each of the four blending components, the accuracy of the PLSR model constructed in the NIR increased compared to that before the removal of the anomalous samples. For the target variable tobacco silk, the R^2^ of the NIR-PLSR model was improved from 0.7717 to 0.8143, and the prediction accuracy was higher than that of the PLSR model constructed by Vis-NIR and fusion spectroscopy. For the other three target variables, the accuracy of the NIR-PLSR model after removing the abnormal samples, although improved, was still not as good as the prediction accuracy of the PLSR model constructed by fusion spectroscopy. For the fusion spectra of Vis-NIR and NIR dataset, the performance of the PLSR model for the fusion spectra in **Table 3** is consistent with that in **Table 2** because the MCD does not exclude the anomalous samples. Overall, when the target variable was tobacco silk, the modeling of NIR was better than Vis-NIR and the fusion spectra of Vis-NIR and NIR; when the target variables were cut stem, fermented cut stem, and expanded tobacco silk, the modeling accuracies of the fusion spectra of Vis-NIR and NIR were the best among the three spectra.

### 3.3 Spectral data processing

#### 3.3.1 Spectral processing results

**Figure 5:**
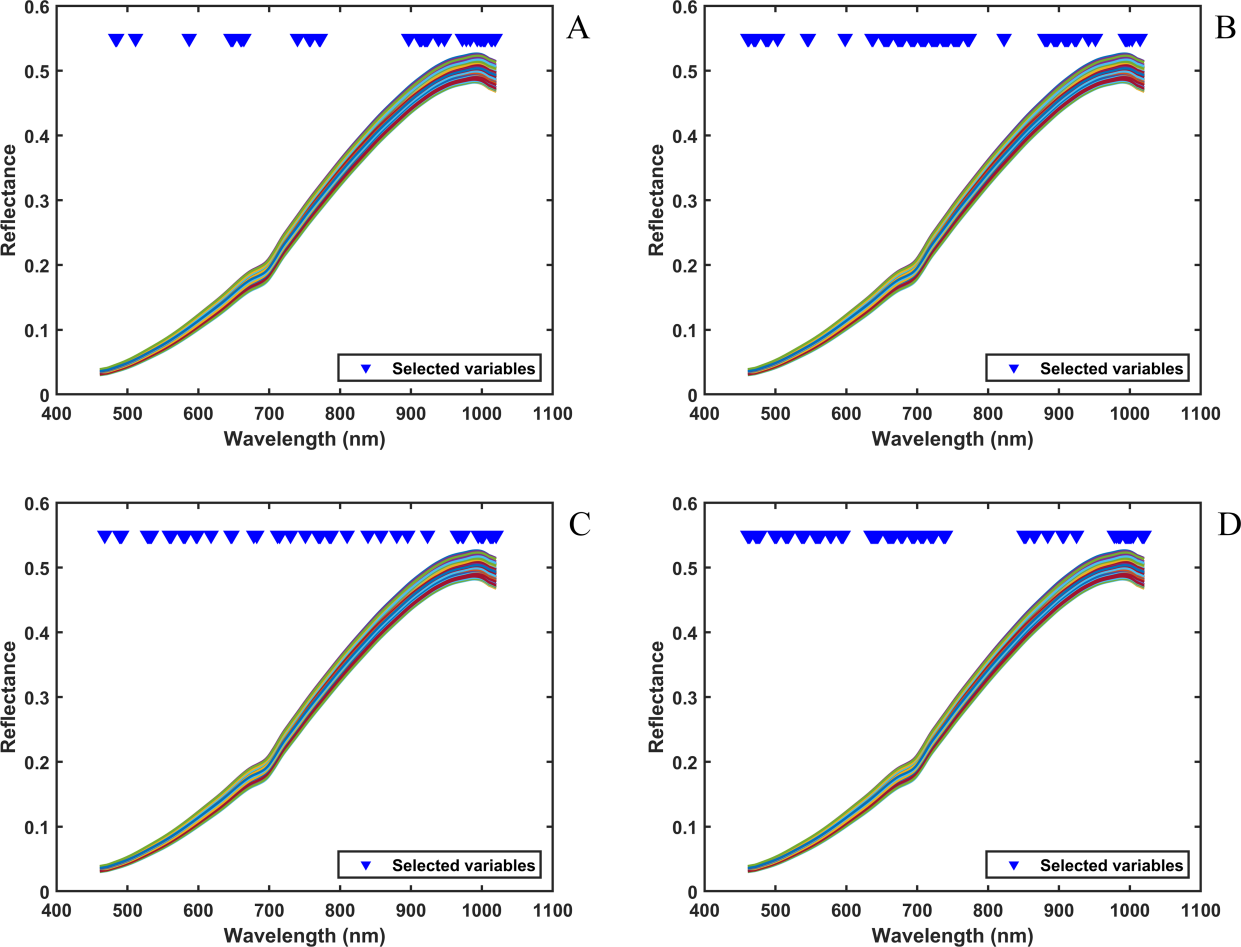
Vis-NIR processed with different blending components: **(A)** tobacco silk; **(B)** cut stem; **(C)** fermented cut stem; **(D)** expanded tobacco silk. The x-axis represents wavelength (nm) and the y-axis indicates reflectance.

The feasibility of enhancing model performance through spectral processing was further investigated. In this study, a range of processing methods provided by the AutoPF framework were implemented on the spectral data for various tobacco components detection to attain optimal PLSR regression outcomes. **Figure 5** presents the processed Vis-NIR spectra when four different tobacco adulterants were used as target variables. The Vis-NIR spectral data were preprocessed using moving average smoothing, a moving average smoothing method that filters out high-frequency noise fluctuations in Vis-NIR by calculating the mean value of the spectral points within a window. By comparing different spectral feature wavelength selection methods, CARS was used to select spectral feature wavelengths in Vis-NIR, and wavelengths with strong correlation with the target variables were filtered to improve the model performance. The blue lower triangles in **Figure 5** indicate the spectral wavelength points selected by CARS. When the target variable was tobacco silk (**Figure 5A**), CARS selected 37 of the 400 wavelength points in Vis-NIR, and the spectral wavelength range was concentrated at 896–1018 nm, which indicated that this spectral wavelength region had a strong correlation with tobacco silk. Similarly, when the target variables were cut stem (**Figure 5B**) and expanded tobacco silk (**Figure 5D**), CARS selected 88 wavelength points in Vis-NIR, and the strong correlation spectral wavelength ranges of cut stem were mainly concentrated in the regions of 636.18-773.49 nm and 892–951 nm, while the strong correlation spectral wavelength ranges of expanded tobacco silk were concentrated in the region of 461–595 nm, 461–595 nm, 634–739 nm and 978–1020 nm regions. In **Figure 5C**, it can be seen that the distribution of the 51 wavelength points selected by CARS for the target variable of fermented cut stem is very uniform. This may be related to the mechanism of the CARS algorithm, where multiple random samples in CARS result in the differences in wavelength distributions retained in different subsets being averaged out. And at the same time, the adjacent wavelengths of Vis-NIR usually have a high degree of multiple covariance, and CARS may retain multiple wavelengths with multicollinearity wavelengths to compensate for the loss of information, so that the selected wavelength points are evenly distributed in the spectrum.

**Figure 6:**
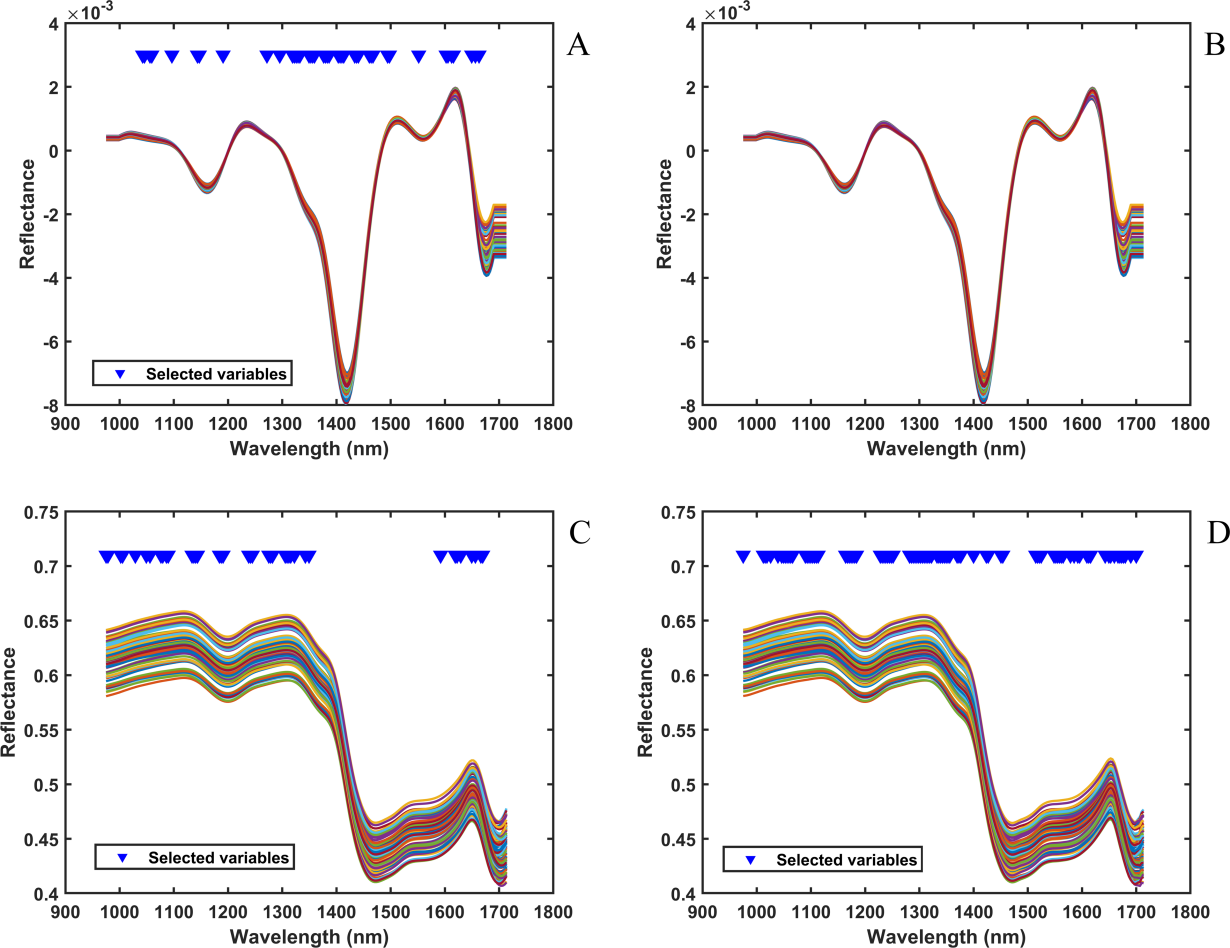
NIR processed with different blending components: **(A)** tobacco silk; **(B)** cut stem; **(C)** fermented cut stem; **(D)** expanded tobacco silk. The x-axis represents wavelength (nm) and the y-axis indicates reflectance.

**Figure 6** shows the NIR after processing for different blending components. When the blending components tobacco silk (**Figure 6A**) and cut stem (**Figure 6B**) were used as the target variables, moving average smoothing and first-order derivative were used as the spectral preprocessing combinations. The first-order derivative effectively separates the overlapping peaks and highlights the weakly absorbing peaks by magnifying the change in slope of the spectral curves. In **Figure 6C**, when the target variable is fermented cut stem, SG smoothing was used as the spectral preprocessing method, and SG smoothing retained the peak shape and amplitude characteristics of the spectra while denoising by polynomially fitting the data points within the window. When the target variable was expanded tobacco silk, as shown in **Figure 6D**, the best regression accuracy could be obtained without the preprocessing method, and thus no preprocessing was performed on the NIR. Subsequently, the 220 spectral wavelength points of NIR were selected for the characteristic wavelengths. For the target variable cut stem (**Figure 6B**), the regression accuracy of the PLSR model was not improved after variable selection, so the full spectrum was modeled using the full spectrum without any variable selection method. After comparing different variable selection methods, CARS was used as the spectral characteristic wavelength selection method for NIR for different blending components. When the target variable was tobacco silk, as shown in Figure A, the 43 wavelength points selected by CARS were mainly concentrated in 1271–1497 nm, which indicated that the wavelength points in this region were strongly correlated with tobacco silk. In **Figure 6C** and **Figure 6D**, it can be seen that the 39 and 93 wavelength points selected by CARS are uniformly distributed in the spectral wavelengths. It is worth noting that when the target variable is fermented cut stem (**Figure 6C**), CARS did not select the wavelength points in the region of 1350–1590 nm, which indicates that the wavelength points in this region of the spectrum are irrelevant wavelengths in predicting fermented cut stem, and do not contain valid information.

**Figure 7:**
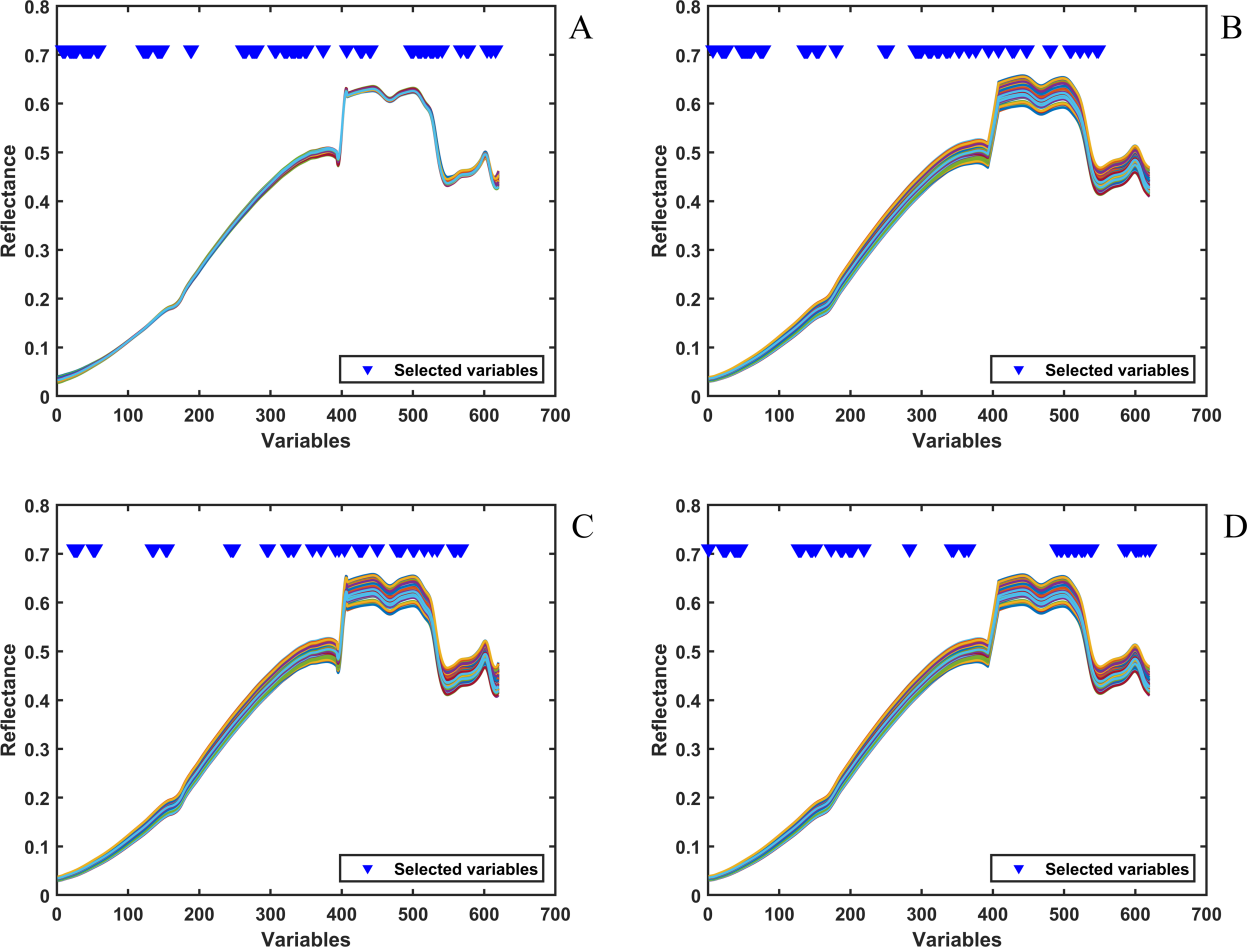
Vis-NIR and NIR fusion spectra processed with different blending components: **(A)** tobacco silk; **(B)** cut stem; **(C)** fermented cut stem; **(D)** expanded tobacco silk. The x-axis represents wavelength (nm) and the y-axis indicates reflectance.

For the fused spectra of Vis-NIR and NIR, feature wavelength selection was performed after preprocessing to enhance model performance as well. In this study, the full spectra of Vis-NIR and NIR were used for spectral fusion, and due to the overlapping bands of Vis-NIR and NIR, variables were used to replace the spectral wavelength points in **Figure 7**, and the total number of variables for the fused spectra was 620.

When the target variable was tobacco silk, the spectra were preprocessed using SG smoothing and MSC to obtain the spectral data in **Figure 7A**. By establishing a linear relationship between the sample spectrum and the reference spectrum, MSC corrects for multiplicative scattering interferences caused by uneven distribution of particles or differences in optical ranges, and can eliminate baseline shifts due to variations in the intensity of the light source or differences in the thickness of the sample. The preprocessing methods were selected based on the prediction accuracy of the model under different preprocessing methods, and the spectral data were preprocessed using moving average smoothing when the target variables were cut stem and expanded tobacco silk, whereas SG smoothing was used as the spectral preprocessing method when the target variable was fermented cut stem. After comparing the different variable selection methods, CARS was used as the feature wavelength selection method for the fusion spectra of Vis-NIR and NIR. In **Figure 7A**, it can be seen that the 135 variables selected by CARS were more evenly distributed, and some wavelength points in both Vis-NIR and NIR were selected for subsequent modeling. In contrast, in **Figure 7B** and **Figure 7C**, CARS did not select variables in the interval [570, 620], and these unselected variables corresponded to 1538–1713 nm in NIR. However, when performing variable selection in NIR only, CARS chose wavelength points in this region to participate in the modeling, which may be due to the fact that some of the bands in Vis-NIR may carry information more directly related to the targeted variables more directly relevant information, leading CARS to prioritize these bands over the 1538–1713 nm band in the fusion spectra of Vis-NIR and NIR. When the target variables were cut stem and fermented cut stem, CARS selected 95 and 60 variables in the fusion spectra of Vis-NIR and NIR, respectively. In **Figure 7D**, when the target variable was expanded tobacco silk, CARS selected 75 out of 620 variables, and the selected variables were mainly concentrated in the intervals [21, 46], [127, 203], [490, 539], and [585, 614], which corresponded to the Vis-NIR’s 489–524 nm and 637–744 nm for Vis-NIR and 1275–1440 nm and 1595–1693 nm for NIR, which indicates that CARS well combines the information from the fusion spectra of Vis-NIR and NIR for subsequent quantitative analysis.

The enhanced performance of the fusion model stems from the complementary information provided by Vis-NIR and NIR spectroscopy. Vis-NIR spectra (400–1000 nm) are primarily sensitive to electronic transitions, offering information related to colorants, pigments, and certain organic functional groups. In contrast, NIR spectra (1000–1700 nm) are dominated by overtone and combination vibrations of fundamental molecular bonds, providing direct insights into the molecular composition, such as moisture, cellulose, nicotine, and other organic constituents. This complementarity is clearly reflected in the feature selection results and detection outcomes. For instance, in the quantitative analysis of fermented cut stem and expanded tobacco silk, the fusion model achieved higher accuracy compared to single-spectrum models. The improvement can be attributed to the fact that these components involve both physical–structural attributes and chemical composition differences. Vis-NIR contributes to correcting scatter effects and detecting color-related changes, while NIR captures detailed chemical vibrations. The fusion strategy thus enables a more holistic characterization, effectively suppressing noise, enhancing feature robustness, and improving predictive reliability—particularly for components with complex or overlapping spectral signatures.

#### 3.3.2 Quantitative analysis results after spectral processing

After outlier removal and processing of Vis-NIR, NIR, and fusion spectra of Vis-NIR and NIR, the prediction results of the PLSR model are shown in **Table 4**.

**Table 4:** Results of PLSR models after spectral processing.

Doping component	Spectral data	Sample size	Number of variables	LVs	R^2^	RMSECV	RPD
Tobacco silk	Vis-NIR	85	37	19	0.8323	0.0281	2.4565
	NIR	84	43	16	0.8442	0.0282	2.5484
	Vis-NIR+NIR	**90**	**135**	**19**	**0.8873**	**0.0236**	**2.9948**
Cut stem	Vis-NIR	85	88	17	0.6272	0.0342	1.6476
	NIR	84	220	12	0.6036	0.0360	1.5979
	Vis-NIR+NIR	**90**	**95**	**12**	**0.6279**	**0.0339**	**1.6466**
Fermented cut stem	Vis-NIR	85	51	11	0.7249	0.0256	1.9179
	NIR	**84**	**39**	**14**	**0.8206**	**0.0200**	**2.3751**
	Vis-NIR+NIR	90	60	18	0.8177	0.0208	2.3550
Expanded tobacco silk	Vis-NIR	85	88	18	0.6818	0.0313	1.7833
	NIR	**84**	**93**	**15**	**0.7303**	**0.0286**	**1.9371**
	Vis-NIR+NIR	90	75	15	0.7202	0.0296	1.9012

Bold values indicate the optimal PLSR model within each component.

Comparing the R^2^ and RMSECV in **Tables 3**, **4**, it can be seen that the spectral processing played a great role in effectively improving the prediction accuracy of the PLSR model. In **Table 4**, when the target variable was tobacco silk, the fusion spectral modeling of Vis-NIR and NIR had the highest prediction accuracy, and the R^2^ was improved from 0.7727 of the original spectra to 0.8873, which was 14.83%. The RMSECV decreased from 0.0334 to 0.0236, representing a relative reduction of 29.34%. Similarly, when the target variable was cut stem, the R^2^ value in the original spectrum increased from 0.5774 to 0.6279, an improvement of 8.75%, and the RMSECV decreased from 0.0361 to 0.0339. This indicates that the fusion of Vis-NIR and NIR spectra significantly improved the performance of quantitative analysis, outperforming single-spectrum models. When the target variables was fermented cut stem and expanded tobacco silk, the NIR model achieved the highest prediction accuracy. The R^2^ values increased from 0.6567 and 0.6120 in the original spectra to 0.8206 and 0.7303, representing improvements of 24.96% and 19.33%, respectively. The RMSECV decreased from 0.0286 and 0.0349 to 0.0200 and 0.0286, representing relative reductions of 30.07% and 18.05%, respectively. For the blending components fermented cut stem and expanded tobacco silk, the modeling accuracies of the fused spectra were slightly lower than those achieved by the NIR model alone. This phenomenon may be attributed to two main factors. First, the spectral response intensity of cut stem in the NIR region is inherently weaker than in the Vis-NIR range, which reduces its contribution to the fused feature set. Second, the overlapping spectral bands between Vis-NIR and NIR in the 975–1020 nm region carry similar chemical information but with different signal-to-noise ratios, which may introduce redundant or conflicting spectral features after fusion, thereby hindering the model’s ability to extract key discriminative signals.

Compared to previous studies, the present work employed hyperspectral imaging technology, which allows for the flexible selection of ROI and enables the fusion of Vis-NIR and NIR spectra. This approach provides a more tailored and potentially more accurate detection method for tobacco shred analysis.

To address this issue and improve fusion performance, several targeted strategies could be explored in future work. For instance, weighted band fusion could be applied to adjust the contribution of NIR features based on their relevance to the target component, rather than treating all wavelengths equally. Alternatively, targeted band screening could be implemented prior to fusion, such as removing the overlapping NIR region or applying segmental noise filtering to the NIR spectrum, to enhance the signal quality and relevance of the fused spectral dataset.

## 4 Conclusion

This study successfully demonstrates that feature-level fusion of Vis-NIR and NIR spectroscopy offers a substantial improvement over single-spectrum methods for predicting the composition content of finished tobacco products. The fused-spectrum PLSR model achieved significantly higher predictive accuracy, with R² increasing from 0.7727 to 0.8873—a relative improvement of 14.83%. This enhancement stems from the complementary information captured by each spectral range. Through feature optimization, the fusion process also effectively suppressed spectral noise and improved model robustness, confirming the clear advantage of multi-spectral analysis for this complex matrix.

These findings carry both theoretical and practical significance. Theoretically, they validate spectral fusion as an effective strategy to overcome the inherent limitations of individual spectroscopic techniques. Practically, the developed model provides the tobacco industry with a rapid, non-destructive tool for precise blend monitoring, which is crucial for maintaining consistent product quality and optimal combustion characteristics.

It is important to acknowledge certain limitations of the current work. The model was developed using a limited sample set from a specific production context, and its evaluation relied on cross-validation rather than external testing, which may affect its generalizability to other tobacco varieties or production batches. Furthermore, spectral acquisition was conducted offline under controlled conditions; implementing this method for real-time, in-line monitoring would require substantial engineering optimization to address challenges such as varying sample presentation and environmental interference on active production lines.

Future research should therefore focus on validating the model with larger and more diverse external sample sets, optimizing the computational workflow for real-time industrial deployment, and exploring the integration of additional spectroscopic modalities to expand the analytical scope. Ultimately, this work establishes a robust analytical framework that provides both methodological insights and a practical foundation for advancing toward accurate, non-destructive quality monitoring in tobacco production and similar blending processes.

## Data Availability

The raw data supporting the conclusions of this article will be made available by the authors, without undue reservation.
