# Supplementary material for: Quantitative analysis of tobacco blending proportions based on hyperspectral imaging and data fusion
Source: Front Plant Sci. 2026 Jan 15;16:1736546. doi: 10.3389/fpls.2025.1736546 (PMC12852377; doi:10.3389/fpls.2025.1736546)
Supplement: Supplementary file 1 [file Table1.docx]

Table S1. Variance contribution rates of the first five principal components for Vis–NIR, NIR, and fused spectra

| Spectral type | Number of  principal components | Variance  contribution rate | Cumulative variance  contribution rate |
| --- | --- | --- | --- |
| Vis-NIR | 1 | 97.4820 | 97.4820 |
|  | 2 | 2.1976 | 99.6797 |
|  | 3 | 0.2701 | 99.9497 |
|  | 4 | 0.0252 | 99.9750 |
|  | 5 | 0.0115 | 99.9865 |
| NIR | 1 | 97.4013 | 97.4013 |
|  | 2 | 1.7076 | 99.1089 |
|  | 3 | 0.6165 | 99.7254 |
|  | 4 | 0.1984 | 99.9238 |
|  | 5 | 0.0523 | 99.9761 |
| Vis-NIR+NIR | 1 | 93.2109 | 93.2109 |
|  | 2 | 4.4777 | 97.6885 |
|  | 3 | 0.9853 | 98.7738 |
|  | 4 | 0.7083 | 99.3821 |
|  | 5 | 0.3557 | 99.7378 |

Table S2. Comparison of PLSR performance using different wavelength selection methods on the Vis–NIR spectra for four tobacco components

| Doping  component | Wavelength  selection methods | Number  of variables | LVs | R^2^ | RMSECV | RPD |
| --- | --- | --- | --- | --- | --- | --- |
| Tobacco silk | None**^1^** | 400 | 10 | 0.7820 | 0.0321 | 2.1544 |
|  | CARS | 37 | 19 | **0.8323** | **0.0281** | **2.4565** |
|  | VIP | 18 | 8 | 0.8015 | 0.0306 | 2.2577 |
|  | SPA | 21 | 16 | 0.7831 | 0.0320 | 2.1599 |
| Cut stem | None | 400 | 10 | 0.5693 | 0.0368 | 1.5328 |
|  | CARS | 88 | 17 | **0.6272** | **0.0342** | **1.6476** |
|  | VIP | 20 | 7 | 0.5999 | 0.0354 | 1.5903 |
|  | SPA | 12 | 10 | 0.5594 | 0.0372 | 1.5155 |
| Fermented cut stem | None | 400 | 14 | 0.7004 | 0.0267 | 1.8377 |
|  | CARS | 51 | 11 | **0.7249** | **0.0256** | **1.9179** |
|  | VIP | 10 | 9 | 0.7065 | 0.0264 | 1.8569 |
|  | SPA | 11 | 10 | 0.6838 | 0.0274 | 1.7890 |
| Expanded tobacco silk | None | 400 | 12 | 0.6023 | 0.0350 | 1.5951 |
|  | CARS | 88 | 18 | **0.6818** | **0.0313** | **1.7833** |
|  | VIP | 20 | 9 | 0.6663 | 0.0321 | 1.7413 |
|  | SPA | 24 | 7 | 0.5668 | 0.0366 | 1.5284 |

1: Only performed spectral preprocessing without wavelength selection.

Table S3. Comparison of PLSR performance using different wavelength selection methods on the NIR spectra for four tobacco components

| Doping  component | Wavelength  selection methods | Number  of variables | LVs | R^2^ | RMSECV | RPD |
| --- | --- | --- | --- | --- | --- | --- |
| Tobacco silk | None | 220 | 16 | 0.8049 | 0.0316 | 2.2776 |
|  | CARS | 43 | 16 | **0.8442** | **0.0282** | **2.5484** |
|  | VIP | 18 | 7 | 0.7877 | 0.0329 | 2.1831 |
|  | SPA | 17 | 16 | 0.8433 | 0.0283 | 2.5413 |
| Cut stem | None | **220** | **12** | **0.6036** | **0.0360** | **1.5979** |
|  | CARS | 12 | 6 | 0.5914 | 0.0366 | 1.5737 |
|  | VIP | 10 | 6 | 0.5749 | 0.0373 | 1.5430 |
|  | SPA | 19 | 7 | 0.5188 | 0.0397 | 1.4502 |
| Fermented cut stem | None | 220 | 19 | 0.7573 | 0.0232 | 2.0419 |
|  | CARS | 39 | 14 | **0.8206** | **0.0200** | **2.3751** |
|  | VIP | 18 | 8 | 0.7673 | 0.0227 | 2.0857 |
|  | SPA | 21 | 11 | 0.7176 | 0.0250 | 1.8931 |
| Expanded tobacco silk | None | 220 | 10 | 0.6408 | 0.0331 | 1.6785 |
|  | CARS | 93 | 15 | **0.7303** | **0.0286** | **1.9371** |
|  | VIP | 18 | 8 | 0.7152 | 0.0294 | 1.8850 |
|  | SPA | 15 | 13 | 0.7220 | 0.0291 | 1.9080 |

Table S4. Comparison of PLSR performance using different wavelength selection methods on the fused spectra of Vis-NIR and NIR for four tobacco components

| Doping  component | Wavelength  selection methods | Number  of variables | LVs | R^2^ | RMSECV | RPD |
| --- | --- | --- | --- | --- | --- | --- |
| Tobacco silk | None | 620 | 15 | 0.7405 | 0.0357 | 1.9739 |
|  | CARS | 135 | 19 | **0.8873** | **0.0236** | **2.9948** |
|  | VIP | 10 | 7 | 0.7832 | 0.0327 | 2.1595 |
|  | SPA | 19 | 9 | 0.7165 | 0.0374 | 1.8887 |
| Cut stem | None | 620 | 15 | 0.6064 | 0.0348 | 1.6029 |
|  | CARS | 95 | 12 | **0.6279** | **0.0339** | **1.6466** |
|  | VIP | 9 | 8 | 0.5242 | 0.0383 | 1.4578 |
|  | SPA | 19 | 9 | 0.4211 | 0.0422 | 1.3216 |
| Fermented cut stem | None | 620 | 17 | 0.7246 | 0.0256 | 1.9162 |
|  | CARS | 60 | 18 | **0.8177** | **0.0208** | **2.3550** |
|  | VIP | 18 | 8 | 0.7757 | 0.0231 | 2.1234 |
|  | SPA | 17 | 7 | 0.5235 | 0.0336 | 1.4568 |
| Expanded tobacco silk | None | 620 | 19 | 0.6387 | 0.0336 | 1.6730 |
|  | CARS | 75 | 15 | **0.7202** | **0.0296** | **1.9012** |
|  | VIP | 10 | 9 | 0.5989 | 0.0354 | 1.5878 |
|  | SPA | 21 | 11 | 0.6023 | 0.0353 | 1.5946 |

Table S5. Performance of PLSR models using fused spectra with and without removal of the overlapping band (975–1020 nm) for different tobacco components

| Doping component | Number of variables | LVs | R^2^ | RMSECV | RPD |
| --- | --- | --- | --- | --- | --- |
| Tobacco silk | 620**^1^** | 18 | **0.7727** | **0.0334** | **2.1093** |
|  | 587**^2^** | 17 | 0.7722 | 0.0335 | 2.1071 |
|  | 606**^3^** | 18 | 0.7654 | 0.0340 | 2.0762 |
| Cut stem | 620 | 10 | 0.5774 | 0.0361 | 1.5469 |
|  | 587 | 9 | **0.5783** | **0.0360** | **1.5486** |
|  | 606 | 16 | 0.5701 | 0.0364 | 1.5337 |
| Fermented cut stem | 620 | 14 | **0.7046** | **0.0265** | **1.8503** |
|  | 587 | 12 | 0.6946 | 0.0269 | 1.8196 |
|  | 606 | 14 | 0.7002 | 0.0267 | 1.8365 |
| Expanded tobacco silk | 620 | 19 | **0.6676** | **0.0323** | **1.7442** |
|  | 587 | 17 | 0.6555 | 0.0328 | 1.7134 |
|  | 606 | 19 | 0.6533 | 0.0330 | 1.7078 |

1: The full fused spectra;

2: Spectra with the overlapping region removed from Vis–NIR;

3: Spectra with the overlapping region removed from NIR.
